# Supplementary figures and images for: Porcine Astrovirus Infection in Brains of Pigs in Korea
Source: Viruses. 2024 Aug 28;16(9):1372. doi: 10.3390/v16091372 (PMC11435919; doi:10.3390/v16091372)

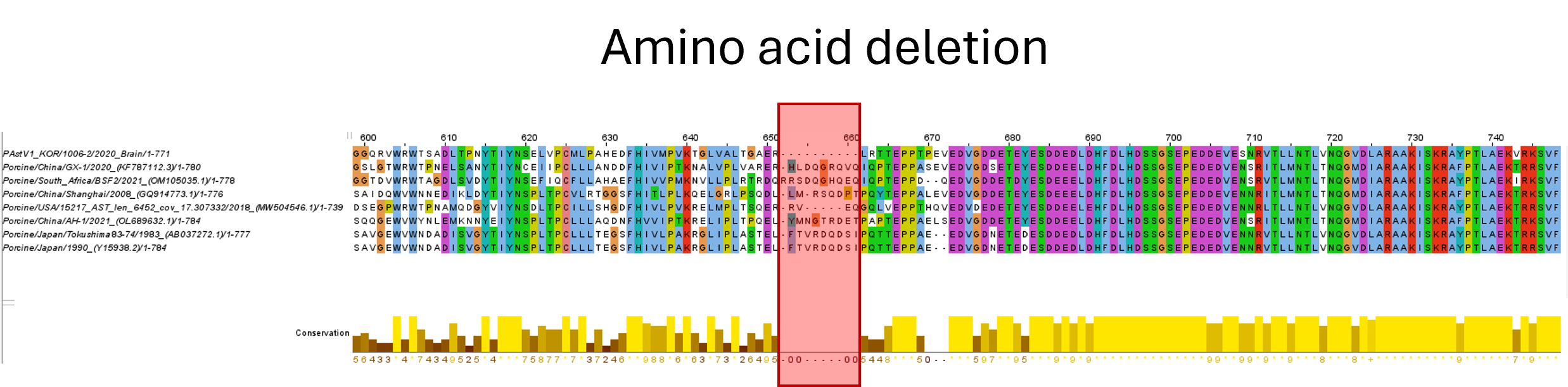

Supplement: Supplementary file 1 [file viruses-16-01372-s001.zip › Supplementary Figure 1 - PAstV1 ORF2 amino acid.png]

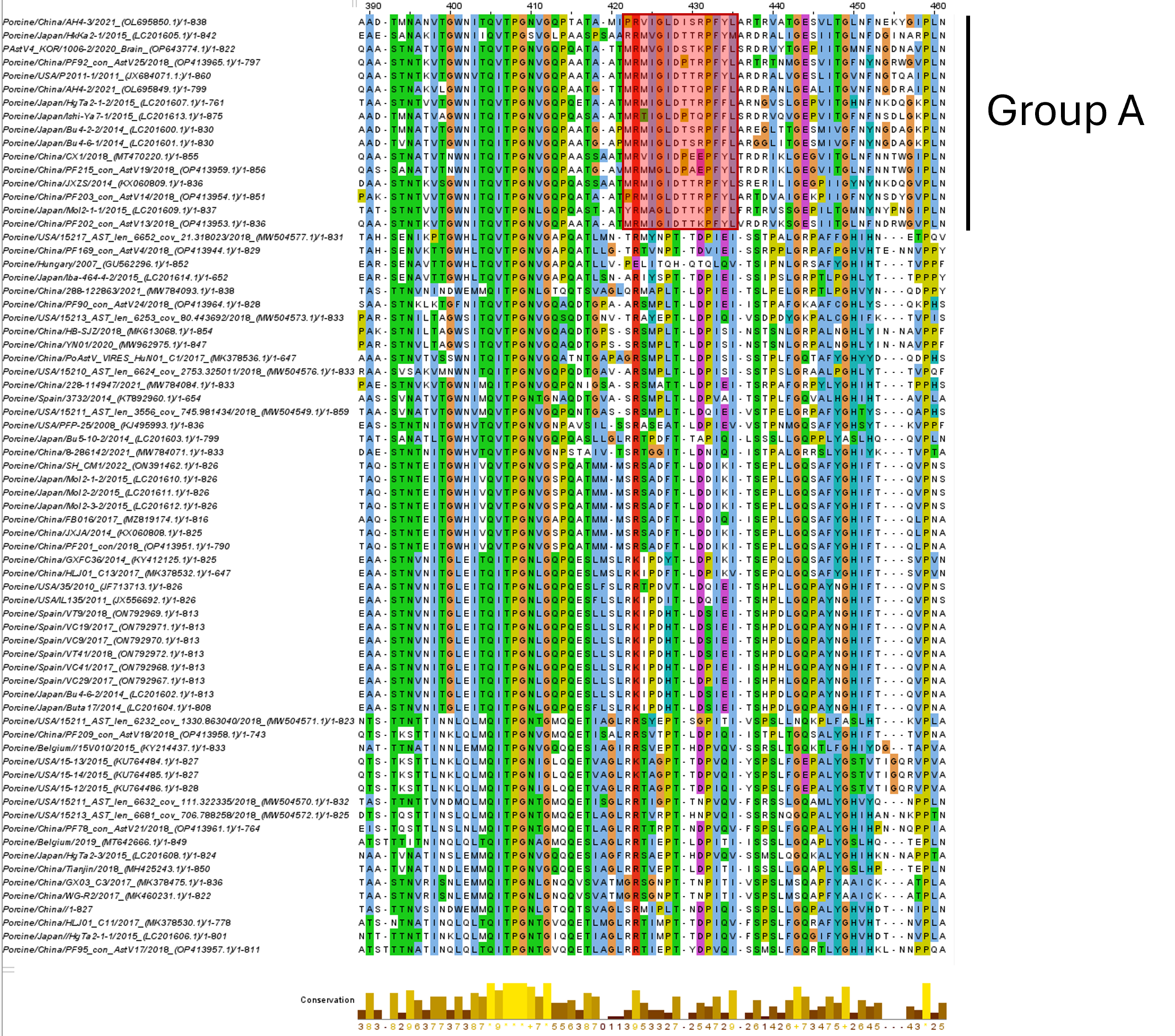

Supplement: Supplementary file 1 [file viruses-16-01372-s001.zip › Supplementary Figure 2 - PAstV4 ORF2 amino acid.png]

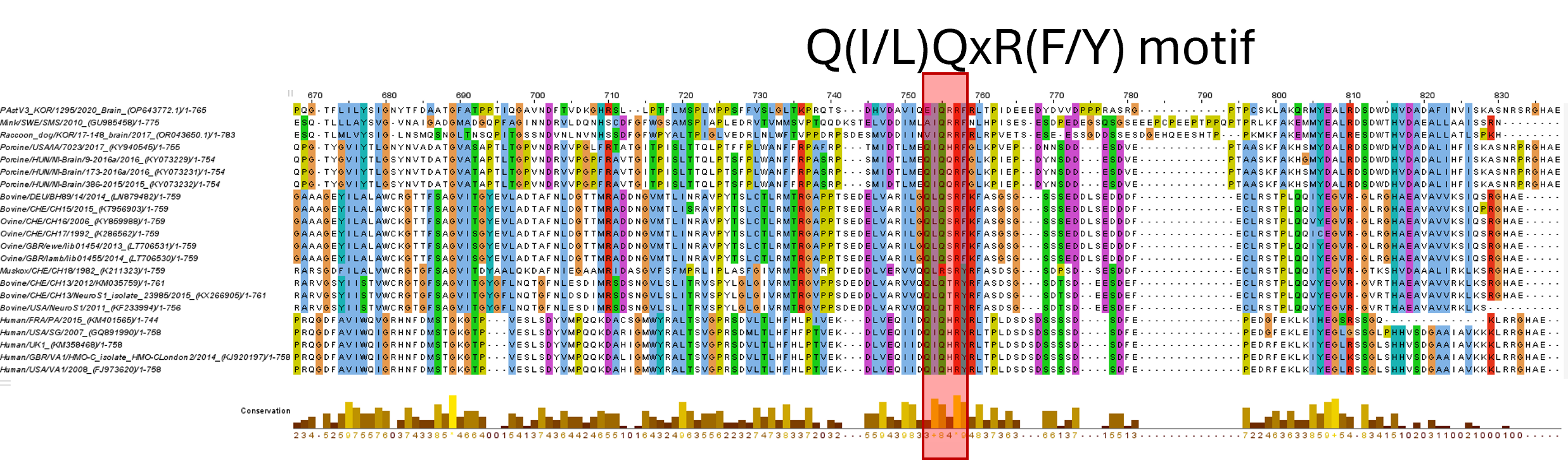

Supplement: Supplementary file 1 [file viruses-16-01372-s001.zip › Supplementary Figure 3 - Ni-AstVs amino acid.png]
